# Simulating the Evolution of von Neumann Entropy in Black Hole Hawking Radiation Using Biphoton Entanglement

**DOI:** 10.3390/e27030236

**Published:** 2025-02-25

**Authors:** Zhuoying Li, Haoshen Fan, Xingwen Zhao, Qinfei Wu, Ji Bian, Yang Liu, Le Luo

**Affiliations:** 1 School of Physics and Astronomy, Sun Yat-Sen University, Zhuhai 519082, China; lizhy379@mail2.sysu.edu.cn (Z.L.); fanhsh5@mail2.sysu.edu.cn (H.F.); zhaoxw@mail.ustc.edu.cn (X.Z.); wuqf8@mail.sysu.edu.cn (Q.W.); bianji@ustc.edu.cn (J.B.); 2 Quantum Science Center of Guangdong-HongKong-Macao Greater Bay Area, Shenzhen 518048, China; 3 Shenzhen Research Institute, Sun Yat-Sen University, Shenzhen 518057, China; 4 Guangdong Provincial Key Laboratory of Quantum Metrology and Sensing, Sun Yat-Sen University, Zhuhai 519082, China; 5 State Key Laboratory of Optoelectronic Materials and Technologies, Sun Yat-Sen University, Guangzhou 510275, China

**Keywords:** black hole information paradox, Page curve, two-photon entanglement, tomography, von Neumann entropy

## Abstract

Addressing the black hole information paradox necessitates the exploration of various hypotheses and theoretical frameworks. Among these, the proposition to utilize quantum entanglement, as introduced by Don N. Page, shows great promise. This study builds upon Page’s theoretical foundation and proposes a simplified model for elucidating the evolution of black hole von Neumann entropy. This model simulates the process of Hawking radiation using entangled photon pairs. Our experiment suggests that quantum entanglement may offer a plausible avenue for resolving the paradox, thereby lending support to Page’s proposal. The results suggest that this model may contribution to the exploration of one of the most profound puzzles in theoretical physics.

## 1. Introduction

The space–time curvature [1] and the no-hair theorem of black holes [2] indicate that any matter crossing the event horizon becomes irretrievably trapped, with its information disappearing. Only three properties are retained by the black hole: mass, charge, and angular momentum. This characteristic, however, appears to conflict with the second law of thermodynamics, as the infall of high-entropy matter into a black hole would seemingly result in a net decrease in the entropy of the universe. Jacob D. Bekenstein addressed this paradox by proposing that black holes have entropy, which is directly proportional to the surface area of their event horizon [3,4]. Meanwhile, Stephen Hawking expanded on this concept by introducing the theory of black hole radiation, which demonstrated that black holes emit thermal radiation [5]. Although Hawking radiation has not been directly observed, recent progress in using the Bose–Einstein condensate of ultra-cold atoms to simulate acoustic black holes has verified Hawking radiation to some extent [6], preliminarily affirming the correctness of Hawking’s theory of radiation. Hawking radiation implies that evaporating black holes emit purely thermal radiation, which carries no quantum information. If a black hole undergoes complete evaporation, the information associated with the matter that entered it would be irretrievably lost. This outcome conflicts with the fundamental principle of information conservation in quantum mechanics, leading to what is known as the black hole information paradox. The black hole information paradox reveals the potential conflict between general relativity and quantum mechanics under extreme gravitational conditions, involving fundamental issues such as quantum information conservation, black hole thermodynamics, and the second law of thermodynamics. Resolving this paradox not only constitutes a crucial test of black hole thermodynamics and the principle of quantum information conservation but also carries far-reaching implications for exploring new physical theories, deepening our understanding of the fundamental laws of the universe, and developing novel observational and experimental methodologies. Don N. Page proposed that quantum information is encoded in the correlation between radiation particles and the black hole [7,8]. He presented the general evolution process of the radiation entanglement entropy, namely the Page curve. Recently, researchers achieved a significant breakthrough by calculating the Page curve using semi-classical techniques, which was accomplished by investigating black holes in asymptotically anti-de Sitter spacetimes connected to a conformal field theory reservoir [9,10]. Moreover, the Island rule formula of Hawking radiation fine entropy was put forward, and the Page curve was derived from the angle of pure gravity [11,12,13]. The challenge of directly observing Hawking radiation makes it difficult to experimentally validate Hawking’s theory. However, recent advancements in gravity and black hole analogues—such as Bose–Einstein condensates [6], optical systems [14,15], and water waves [16]—have provided new insights into black hole dynamics. The aim of this paper is to demonstrate the time-dependent change in entropy of Hawking radiation using a simple and easily verifiable model. This study builds upon Page’s theory by modeling the black hole as a two-photon entangled system. Through a direct calculation of the von Neumann entropy, we derived and experimentally verified the Page curve, illustrating the time evolution of black hole entropy, which initially increases and subsequently decreases back to its initial value during Hawking radiation, thus successfully reproducing the key qualitative features of black hole evaporation and suggesting that unitarity may be preserved in physical black holes. Although the simplified model does not fully capture the complex dynamics of real black holes and has certain limitations in addressing unknown physical phenomena, it offers a valuable perspective for intuitively understanding the black hole information paradox and the process of information transfer, thereby laying a foundation for further research in this field.

## 2. Theory

The solution proposed by Don N. Page regarding Hawking radiation and quantum entanglement suggests that maximally entangled pairs of particles can be viewed as being in a pure state as a whole [17]. However, when this pair of particles is separated, each individual particle will be in a completely mixed state, possessing maximal von Neumann entropy. Vacuum quantum fluctuations near the event horizon of a black hole produce pairs of virtual particles in a maximally entangled state. Under the influence of the black hole’s intense gravitational field, one particle from each pair is captured by the black hole, while the other escapes, transitioning to a real particle and forming what is observed as Hawking radiation. By assuming that the increase in von Neumann entropy of the black hole system arises exclusively from the particle evolution process between the black hole and its Hawking radiation, without external influences, it follows that the maximum von Neumann entropy of the combined black hole and radiation system must be equal to each other. Furthermore, this maximum entropy should not exceed the semi-classical entropy of the system, ensuring consistency with established thermodynamic principles [18].

(1) $$\begin{array}{cc} S_{vN} & =\min\left[{\tilde{S}}_{BH},{\tilde{S}}_{\mathrm{rad}\;}\right]\\ \; & =4\pi M_{0}^{2}\cdot \min\left\{{\left(1-t/t_{\mathrm{decay}\;}\right)}^{2/3},1.48472\left[1-{\left(1-t/t_{\mathrm{decay}\;}\right)}^{2/3}\right]\right\} \end{array}$$

${\tilde{S}}_{BH}$ and ${\tilde{S}}_{\mathrm{rad}\;}$ represent the semi-classical entropies of the black hole and the Hawking radiation, respectively. $M_{0}$ denotes the initial mass of the black hole at time *t* = 0, while $t_{\mathrm{decay}\;}=8895\;M_{0}^{3}$ specifies the decay time for a non-rotating uncharged black hole. The 1.48472 is the ratio of the increasing entropy of the radiating part to the decreasing entropy of the black hole part.

Based on this theory, this paper views the black hole as a system with quantum entanglement properties and directly calculates the evolution of von Neumann entropy over time during the Hawking radiation process using a biphoton entanglement model, without relying on semi-classical entropy calculations. We construct a simplified model of Hawking radiation by assuming a non-rotating uncharged Schwarzschild black hole in flat spacetime. In this model, the event horizon is treated as the radiating surface, emitting photons that follow a blackbody radiation distribution. The black hole evaporation process and Hawking radiation are equated to the gradual separation of N maximally entangled photon pairs. In this process, it is assumed that the radiation preferentially separates unsplit photon pairs until each photon inside the black hole has its entangled partner in the Hawking radiation. The moment when this condition is reached can be considered equivalent to the Page time.

According to the von Neumann entropy formula for black holes given in Ref. [19] (see Equation (6.2)),

(2) $$S={min}_{X}\left\{{\mathrm{ext}}_{X}\left[\frac{\mathrm{Area}(X)}{4G_{N}}+S_{\text{simi-cl}}\left(\Sigma_{X}\right)\right]\right\}.$$

**Figure 1:**
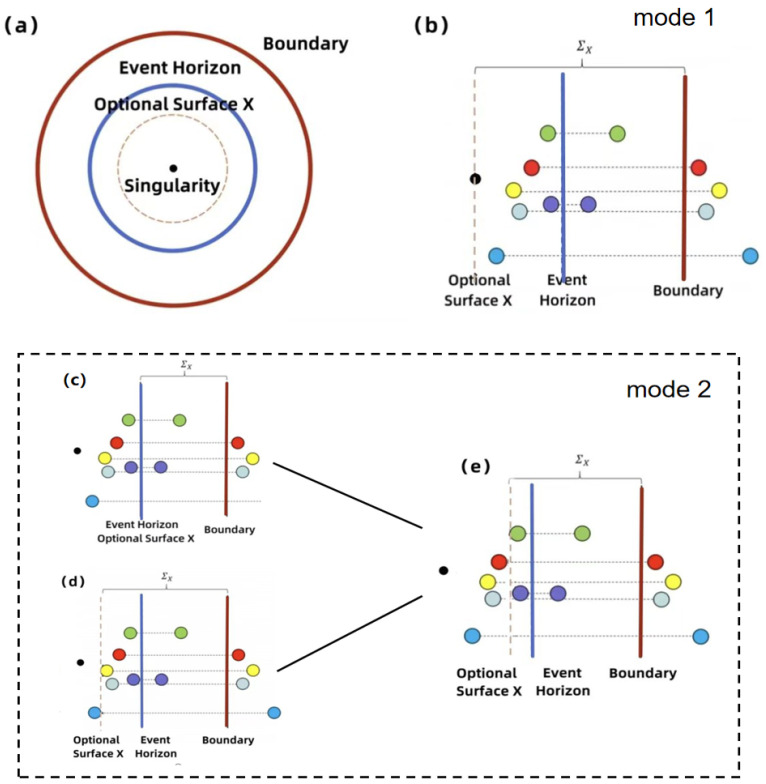
(**a**) Black hole schematic diagram. The red curve and blue curve represent the defined boundary of the black hole and the event horizon, respectively, while the dotted line denotes the optional surface *X*. In this context, the black hole region is characterized as the area confined within the defined boundary. The surface *X* position is adjustable, and the part between the surface *X* and the boundary is referred to as $\Sigma_{X}$. (**b**) Schematic diagram of Mode 1, where the optional surface *X* is located at a singularity. The balls of the same color connected by dotted lines represent a pair of positive and negative particles produced in the vacuum, and each such pair of particles is separated by the event horizon of a black hole. (**c**–**e**) Schematic diagram of Mode 2, where the optional surface *X* is not located at a singularity. (**c**) The optional surface *X* is directly selected at the event horizon. (**d**) The optional surface *X* is too close to the singularity. (**e**) The optional surface is selected at an appropriate position.

We can qualitatively explain the rationality of the ‘splitting’ assumption. From this formula, the rationality of the ‘splitting’ assumption can be qualitatively explained. From this formulation, it is evident that the total entropy of the black hole is composed of two distinct components. The first component, $\frac{\mathrm{Area}(X)}{4G_{N}}$, is associated with the geometric properties of the black hole, commonly understood as its information storage capacity. Therefore, the surface area of the selected surface *X* can be considered as the first contribution to the generalized entropy. The second part $S_{\text{simi-cl}}\left(\Sigma_{X}\right)$ involves quantum entanglement properties, describing the interaction between quantum fields. In the current model, since external factors are not considered, the second contribution comes from the vacuum quantum fields in the region $\Sigma_{X}$, which consist of particle pairs generated by vacuum fluctuations. By adjusting the position of surface *X* and searching for the extremum of the generalized entropy and selecting the smallest value, namely ${min}_{X}\left\{{\mathrm{ext}}_{X}[]\right\}$ (‘ext’ represents taking the extremum of the generalized entropy, and ‘min’ indicates that when there are multiple extremal surfaces, the one that minimizes S is chosen), the von Neumann entropy of the black hole can then be determined. Consequently, a simplified depiction of the black hole structure, illustrated in Figure 1, can be constructed. In the figures, the red solid line is located a few Schwarzschild radii away from the singularity and is defined as the boundary of observation; the blue solid line represents the event horizon, situated between the singularity and the boundary. The position of surface *X* can be freely chosen between the singularity and the event horizon, with the region enclosed between surface *X* and the boundary designated as $\Sigma_{X}$.

Before the black hole begins emitting Hawking radiation, the vacuum quantum field does not contribute to the entropy, and there are no isolated particles in $\Sigma_{X}$. At this stage, the minimum von Neumann entropy corresponds to selecting surface *X* directly at the singularity, with the von Neumann entropy being zero. Therefore, we may initially fix surface *X* at the singularity (as shown in Figure 1b). When Hawking radiation commences, pairs of entangled particles at the event horizon become separated and are emitted in opposite directions, with some particles gradually crossing the boundary and escaping from the black hole, while the correlated entangled particles contained in the region $\Sigma_{X}$ move inward. At this point, the von Neumann entropy inside and outside the black hole increases simultaneously and in equal measure. We define this evolution mode as Mode 1. However, as the radiation process continues, the von Neumann entropy corresponding to surface *X* placed at the singularity may no longer be the minimum. In this case, surface *X* can be moved to the event horizon (as shown in Figure 1c), where it becomes evident that $\Sigma_{X}$ contains only a few radiated particles; so, its von Neumann entropy is not zero. On this basis, if surface *X* is moved further toward the singularity (as shown in Figure 1d), the value of the geometric term decreases, and since it includes entangled pairs of radiated particles, the second term also decreases. Ultimately, the generalized entropy of the entire system decreases and reaches a minimum. We define this evolution mode as Mode 2.

It is worth noting that in Mode 2, the minimum value corresponding to surface *X* is always near the event horizon, and this minimum closely corresponds to the semi-classical entropy of the black hole, commonly referred to as the area entropy. With the progression of the radiation and the evaporation of the black hole, these entropies gradually decrease. Accordingly, the von Neumann entropy of the black hole corresponds to the extremum value obtained from the two previously described modes: the result of Mode 1 starts from zero and continuously increases as radiation proceeds, while the result of Mode 2 gradually decreases to zero, as the black hole evaporates. Taking the minimum of these two values reveals a trend that the von Neumann entropy first increases and then decreases, providing insight into the black hole information paradox. This argument corresponds directly to the ‘splitting’ hypothesis of biphoton entanglement (as shown in Table 1), demonstrating the rationality of the proposed hypothesis.

Returning to the simplified black hole radiation model mentioned earlier, in order to calculate the evolution of the number of radiated particles over time, we use the Unruh Effect to compute the Hawking temperature of the black hole [20], treating the event horizon $\left(r_{0}=2M\right)$ as the radiating surface. From this, we obtain the total energy and total number of particles radiated per unit time [more details in Appendix A]:

(3) $$T\left(\infty \right)=\frac{1}{8\pi M}$$

(4) $$\begin{array}{cc} \; & P=4\pi r_{0}^{2}\cdot p=C_{p}M^{-2}\\ \; & N=4\pi r_{0}^{2}\cdot n=C_{N}M^{-1}. \end{array}$$

The model assumes that the mass loss entirely results from photons radiated outward from the event horizon; thus, according to the mass–energy equation, we can derive the following:

(5) $$P=\frac{\Delta E}{\Delta t}=\frac{-\Delta Mc^{2}}{\Delta t}=-\frac{dM}{dt}=C_{p}M^{-2}.$$

Solving this differential equation yields the relationship between mass and time evolution:

(6) $$M\left(t\right)=M_{0}{\left(1-t/\alpha \right)}^{1/3}$$

(7) $$\alpha =\frac{M_{0}^{3}}{3C_{p}}=5120\pi^{2}M_{0}^{3}.$$

By incorporating the temporal evolution of mass into the expression for the particle number, it can be determined that the variation in the number of particles emitted per unit time over time is

(8) $$N\left(t\right)=C_{N}M_{0}^{-1}{\left(1-t/\alpha \right)}^{-1/3}.$$

Through integrating the above formula with respect to time, we can obtain that the variation of the total particle number of the radiative portion at time *t* with respect to time is

(9) $$N_{\mathrm{radiation}\;}\left(t\right)=\frac{3}{2}\alpha C_{N}M_{0}^{-1}\left(1-{\left(1-t/\alpha \right)}^{2/3}\right).$$

The complete evolution of von Neumann entropy over time is

(10) $$\begin{array}{cc} S_{vN} & =\frac{3}{2}\alpha C_{N}M_{0}^{-1}\cdot \min\left[\left(1-{\left(1-t/\alpha \right)}^{2/3}\right),{\left(1-t/\alpha \right)}^{2/3}\right]\cdot \ln2\\ \; & \approx 3.22464M_{0}^{2}\cdot \min\left[\left(1-{\left(1-t/\alpha \right)}^{2/3}\right),{\left(1-t/\alpha \right)}^{2/3}\right]. \end{array}$$

It is particularly noteworthy that although this model is based on discrete photon pairs, the derived particle number formula (Equation (9)) exhibits qualitative consistency with the semi-classical entropy calculation (Equation (1)). After the Page time, the entropy decrease in both cases originates from the complete separation of entangled pairs. This result indicates that while the discrete model serves as a simplified framework, it effectively captures the universal principles of information transfer in Hawking radiation.

## 3. Experiment

**Figure 2:**
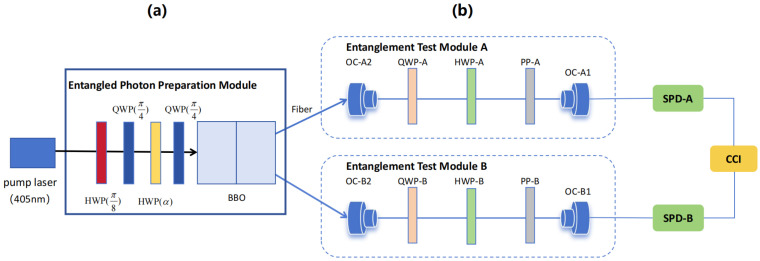
Experimental light path diagram: the left module is the optical path diagram of relative phase modulation and entangled state preparation, and the right module is the entanglement detection optical path diagram. (**a**) The pump laser (405 nm) is adjusted for polarization using wave plates before entering the BBO crystal to generate entangled photon pairs, which are then transmitted to paths A and B. (**b**) Each path contains a QWP and HWP for entanglement testing. A polarizer (PP) selects specific polarization states, and an optical coupler (OC) ensures signal transmission. The entangled photons eventually reach the single-photon detectors (SPD-A and SPD-B), with a coincidence counter (CCI) used to verify the photon entangled state.

Based on the aforementioned theory, we designed the experimental optical setup shown in Figure 2 to verify the biphoton entanglement scheme. As shown in Figure 2a, a pump laser with a wavelength of 405 nm was directed through two half-wave plates (HWP) and two quarter-wave plates (QWP) to precisely adjust and control its polarization state. The adjusted laser then entered a BBO (Beta Barium Borate) crystal, generating entangled photon pairs through the spontaneous parametric down-conversion (SPDC) process. The laser was a 40 mW continuous wave laser with output polarized in the vertical direction. The BBO crystal consisted of two thin BBO crystals with their optical axes perpendicular to each other, bonded together for the type-I parametric down-conversion process. The generated entangled photon pairs passed through a half-wave plate, which adjusted the polarization of each optical path and were transmitted via optical fibers to two independent paths, A and B. Figure 2b illustrates the detailed structure of the two entanglement test paths, A and B. Each path contained a QWP and two HWPs to precisely adjust the polarization state of the photons. A polarizer (PP) was used to select photons with specific polarization states, and an optical coupler (OC) ensured the efficient transmission of the optical signal. The entangled photon pairs entered path A and path B, respectively. After being adjusted by the corresponding optical components, they finally reached single-photon detectors (SPD-A and SPD-B). A coincidence counter (CCI) was employed to evaluate whether the photon detection results from both paths satisfied the coincidence conditions, thus confirming the entangled state of the biphotons.

In this experiment, the black hole was simplified to a system composed of five pairs of maximally entangled photons ($A_{1}$–$B_{1}$, $A_{2}$–$B_{2}$, ……, $A_{5}$–$B_{5}$), generated via SPDC. The black hole evaporation process was entirely framed within the context of quantum optics theory, with the “horizon”—treated as a phenomenological construct corresponding to the optional surface *X*-serves as an operational partitioning tool. This tool divided the observable radiation (the extracted photons) from the simulated black hole microstates (the remaining photons) to characterize the entanglement dynamics between the radiated and residual components. Therefore, the evaporation process was mapped to the gradual evolution of these entangled photon pairs, with data selection performed in accordance with Page’s theory. At five distinct time intervals, we recorded real-time single-photon counts from SPD-A and real-time coincidence counts from the coincidence counter (see Appendix C for details). Among these data, the real-time single-photon counts of SPD-A represented the photons that preferentially escape from the black hole at the initial stage of the Hawking radiation simulation, thereby modeling the “thermal” nature of early black hole radiation. During this stage, the radiation did not carry the black hole’s information, and the number of radiated photons continued to increase until it reached a maximum (five sets of single-photon counts $A_{1}$∼$A_{5}$, corresponding to a 50% loss in black hole mass). Subsequently, photons traveling along path B ($B_{i}$) began to be emitted, simulating the transfer of black hole information to the radiation. In this phase, part of the radiation reconstructed the information correlations via entanglement. This reconstruction was evidenced by the average coincidence count—indicating entanglement reestablishment—and an entanglement fidelity exceeding F > 0.977, which strongly suggest that the process was consistent with unitarity, as no irreversible loss of information was detected within the measured subsystem. As evaporation proceeded, all photons were ultimately radiated away.

By adjusting the half-wave plates and quarter-wave plates to different angles, we changed the measurement basis and used the photon counts in the current basis vectors as direct measurement values to reconstruct the system’s density matrix. In the experiment, H and V corresponded to the horizontal and vertical polarizations, A and D corresponded to the 45° and −45° diagonal polarizations, and L and R corresponded to the left-hand and right-hand circular polarizations. We used different polarizations as basis vectors to measure the single-photon counts in a single channel to characterize the behavior of individual photons. For entangled photon pairs, we selected H, V, D, and R as the bases and constructed 16 basis vector combinations. By recording the coincidence counts in both channels, we reconstructed the density matrix of the biphoton system.

## 4. Results

The density matrix of a single photon can be expressed in its expanded form as [21]

(11) $$\hat{\rho }=\frac{1}{2}\sum_{i=0}^{3}S_{i}{\hat{\sigma }}_{i},$$

where $\sigma_{0}$ represents the identity matrix, and the remaining three are in the order of Pauli x, y, z matrices. The parameter $S_{i}$ is called the Stokes parameter and represents the components of the density matrix on the individual basis vectors, which can be defined by three sets of generally orthogonal measurement bases:

(12) $$\begin{array}{cc} \; & S_{0}=P_{|H\rangle }+P_{|V\rangle }\\ \; & S_{1}=P_{|D\rangle }-P_{|A\rangle }\\ \; & S_{2}=P_{|R\rangle }-P_{|L\rangle }\\ \; & S_{3}=P_{|H\rangle }-P_{|V\rangle }. \end{array}$$

The P here refers to the probability associated with the corresponding measurement basis. For example, the probability for the horizontal polarization state |H〉(or |D〉) is given by $P_{|H\rangle }=\frac{n_{|H\rangle }}{n_{|H\rangle }+n_{|V\rangle }}\left(P_{|D\rangle }=\frac{n_{|D\rangle }}{n_{|D\rangle }+n_{|A\rangle }}\right)$, where $n_{|H\rangle }\left(n_{|D\rangle }\right)$ and $n_{|V\rangle }\left(n_{|A\rangle }\right)$ represent the number of photons measured in the |H〉(|D〉) and |V〉(|A〉) states, respectively. For more details, refer to Appendix C. By measuring the photon counts under the measurement bases H, V, D, A, and L, R and processing the data accordingly, the Stokes parameters can be obtained, which can then be used to reconstruct the density matrix of a single photon $\rho_{p1}$∼$\rho_{p5}$.

For entangled photon pairs, the density matrix is represented as a 4 × 4 matrix. In the space of 4 × 4 Hermitian matrices, a total of 16 basis vectors are required. These basis vectors can be constructed using the pairwise Kronecker product of four Pauli matrices.

(13) $$\hat{\rho }=\frac{1}{4}\sum_{i,j=0}^{3}S_{ij}\left(\sigma_{i}⊗\sigma_{j}\right)$$

The Stokes parameter is satisfied [21]:

(14) $$S_{ij}=\mathrm{Tr}\left(\hat{\rho }\left(\sigma_{i}⊗\sigma_{j}\right)\right).$$

When using different measurement vectors to measure the quantum state, the measurement probability value meets

(15) $$P_{s}=\langle \psi_{s}\left|\rho \right|\psi_{s}\rangle =\frac{1}{4}\sum_{i,j=0}^{3}\langle \psi_{s}\left|S_{ij}\left(\sigma_{i}⊗\sigma_{j}\right)\right|\psi_{s}\rangle .$$

$\psi_{s}\left(s=1∼16\right)$ is a selected set of sixteen measurement vectors. The detailed calculation process of the measurement probability $P_{s}$ (For example, $P_{|HH\rangle }$) can be found in Appendix C.

After measuring the quantum state with 16 measurement vectors, Stokes parameters are written in the matrix form $\hat{S}$ with

(16) $$\hat{P_{s}}=\frac{1}{4}\hat{T}\hat{S},$$

where the matrix entries of a *T* matrix are is $T_{ij}=\langle \psi_{i}\left|\left(\Gamma_{j}\right)\right|\psi_{i}\rangle \left(i,j=1,2,3,\ldots ,16\right)$, with $\Gamma_{1∼16}=\sigma_{0∼3}⊗\sigma_{0∼3}$. $\hat{P_{s}}$ and $\hat{S}$ are column matrices composed of 16 probability values and Stokes parameters.

According to the above formula, the matrix $\hat{S}$ is $\hat{S}=4{\hat{T}}^{-1}\hat{P_{s}}$. The Stokes parameters are subsequently calculated and substituted into Equation (13) to reconstruct the density matrix. However, tomographic measurements of density matrices can occasionally yield results that violate fundamental properties, such as positivity. Specifically, if the density matrix exhibits negative eigenvalues, it becomes impossible to compute the von Neumann entropy. To address this issue, the maximum likelihood estimation (MLE) method [22] is employed to optimize the density matrix $\rho_{e6∼e10}$. For a detailed explanation of the procedure, please refer to Appendix D.

**Figure 3:**
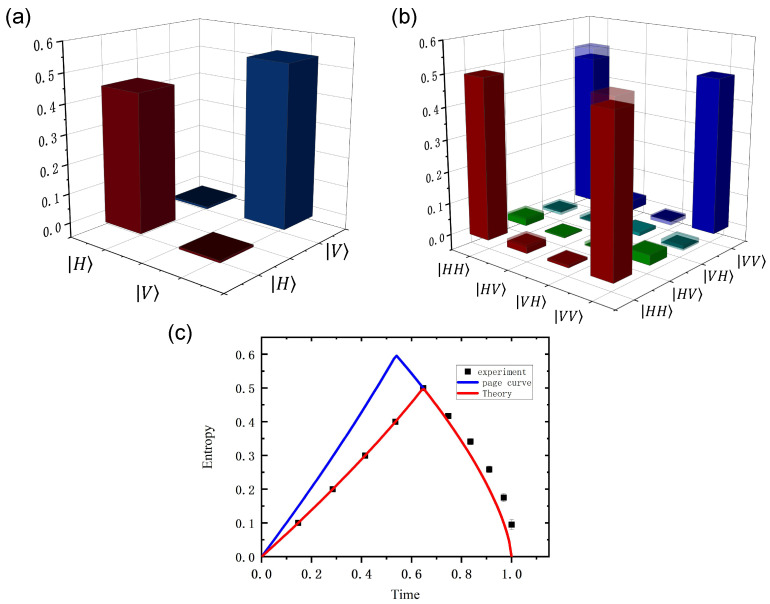
(**a**) The real part of the single-photon state density matrix. (**b**) The real part of the two-photon state density matrix.The semi-transparent region shows the difference between the raw density matrix and the MLE-reconstructed density matrix. (**c**) Evolution of von Neumann entropy over time. The solid red line represents the theoretical prediction (Equation (10)), the solid blue line corresponds to the Page curve (Equation (1)), and the black dots indicate the experimental results. To facilitate a clearer comparison of the von Neumann entropy trends, the entropy values have been normalized.

The density matrices of the single-photon state and two-photon state are shown in Figure 3a and Figure 3b, respectively. In the two-photon density matrix, the real part is the MLE-density matrix, and the virtual part is the raw density matrix. As Hawking radiation progresses, the radiation particles will increase with time, and the density matrix of the first five particles can be expressed as

(17) $$\rho_{n}=\rho_{p1}⊗\rho_{p2}\ldots ⊗\rho_{pn}.$$

Since the first five photons emitted are independent single photons without entanglement, the density matrix at this time is the direct product of the single-photon density matrices. Starting from the radiation of the sixth photon, entangled photon pairs will appear in the radiation section, giving

(18) $$\begin{array}{cc} \rho_{6}= & \rho_{e6}⊗\rho_{p2}⊗\rho_{p3}⊗\rho_{p4}⊗\rho_{p5}\\ \rho_{7}= & \rho_{e6}⊗\rho_{e7}⊗\rho_{p3}⊗\rho_{p4}⊗\rho_{p5}\\ \; & \ldots \\ \rho_{10}= & \rho_{e6}⊗\rho_{e7}⊗\rho_{e8}⊗\rho_{e9}⊗\rho_{e10}. \end{array}$$

By substituting these ten density matrices of the radiation section into the formula $S_{vN}=-\mathrm{Tr}\left[\hat{\rho }log\hat{\rho }\right]$, the corresponding von Neumann entropy can be calculated. Then, according to Equation (9), the corresponding time of the current partial particle number of radiation can be inversely solved, and the change in the von Neumann entropy with time in the process of Hawking radiation can be obtained.

The von Neumann entropy curve plotted from the experimental results is highly consistent with the theoretically predicted curve (as shown in Figure 3c). This consistency demonstrates the reliability of the theoretical derivation and also validates the feasibility of the experimental setup. The result also indicates that during the black hole evaporation process, the quantum information is initially stored in the black. The trend of entropy increasing and then decreasing in the hole is gradually transferred to the radiation through entangled photon pairs in Hawking radiation, which directly reflects the dynamic process of information transfer from the black hole to the radiation.

Entropy increase phase: The internal information of the black hole appears to be “lost” due to the partial separation of entangled photon pairs.

Entropy decrease phase: After the Page time (t≈0.65), the radiation part contains the complete information of the entangled pairs, restoring information conservation.

This result is in agreement with Page’s prediction, which indicates that the process preserves unitarity and also suggests that the dynamic separation mechanism of entangled photon pairs can effectively simulate the resolution of the black hole information paradox.

Since the biphoton entanglement model proposed in this paper cannot consider the scattering effects included in the radiation process of the Page model, the ‘transition time’ of the two curves does not completely coincide. Nevertheless, the similar trend exhibited by both curves is sufficient to demonstrate the validity of this model and further supports the reliability of resolving the black hole information paradox through entanglement theory.

Meanwhile, it is worth noting that there are some experimental errors in the figure. These errors mainly originate from the limitations in the stability of the biphoton entanglement source, as well as systematic errors in the single-photon detectors and optical components. Additionally, fluctuations in the environmental conditions during the experiment also had some impact on the results. Although these errors did not significantly affect the accuracy of the overall trend, it is necessary to further improve the stability of the entanglement source, reduce systematic errors in the detection equipment and optical components, and enhance control over environmental conditions in future experiments to improve the precision and repeatability of the experimental results.

## 5. Conclusions

This paper presented a simulation of the evolution of von Neumann entropy during Hawking radiation using a system of entangled photon pairs. The information paradox arises from the apparent contradiction between the purportedly thermal characteristics of Hawking radiation and the fundamental principle of quantum information conservation. Our experimental framework revealed that through controlled spatial separation and subsequent interference of entangled photon pairs, the quantum state of the radiation subsystem undergoes a transformation sequence, starting from an initial pure state (characterized by low entropy) to a mixed state (exhibiting elevated entropy) and ultimately recovering purity (returning to low entropy). This observation suggests that radiation possessing thermal-like properties may maintain quantum correlations with a black hole’s interior particles. Following complete spatial separation and subsequent recombination of all entangled pairs, the radiation subsystem’s von Neumann entropy approaches the initial value, indicating full information retention during the simulated black hole evaporation process. Therefore, the evolution is consistent with quantum mechanical unitarity principles and can be interpreted as the purification of the localized state via the leakage of photons, whih is central to addressing the black hole information paradox. However, since this evolution process does not provide a comprehensive and detailed simulation of the complete Hawking radiation process, its result should not be regarded as a definitive resolution of the black hole information paradox. To fully resolve the paradox, a more advanced experimental system and more rigorous theoretical support remain necessary. Nonetheless, this experiment clearly indicates that both the transfer of information and the reconstruction of entanglement during the process of black hole Hawking radiation can be effectively explained by fully quantum processes. This result not only deepens our understanding of the mechanisms underlying information conservation in black hole Hawking radiation but also indicates that entangled photon systems can serve as a promising platform for further exploring the fundamental processes in black hole thermodynamics.

In future research, we plan to incorporate an ion–photon entanglement system to further expand the current experimental framework, thereby simulating more complex black hole physics scenarios such as rotating (Kerr) black holes [23], charged (Reissner–Nordstrom) black holes [24], and quantum gravity effects [25,26] under higher-dimensional or dynamically evolving spacetimes, etc. In this ion–photon approach, the trapped ions can serve as the “black hole”, while the photons simulate the “Hawking radiation”. Their respective decoherence and entanglement recovery processes correspond to the apparent information loss and eventual restoration that occur during black hole evaporation. By encoding orbital angular momentum states or introducing effective charge interactions, it becomes possible to model the evolution of spin in a rotating black hole or charge in a charged black hole throughout the evaporation process, and to observe the dynamic transfer of quantum information between the radiated photons and the remaining system. Moreover, by combining high-dimensional entangled-state preparation, multi-modal quantum measurement techniques, and controllable noise channels, one can systematically investigate the impact of quantum fluctuations and spacetime quantization effects (e.g., “spacetime foam”) on information fidelity. The multi-degree-of-freedom controllability and high-precision manipulation afforded by ion–photon systems provide a highly flexible route for simulating the spin, charge, and complex spacetime structures of realistic black holes, offering valuable insights into the mechanisms of Hawking radiation and the black hole information paradox.

## Figures and Tables

**Table 1:** This is a comparison of the two methods.

Two-Photon Model Method	Minimizing Generalized Entropy Method
The von Neumann entropy of the radiation component	Mode 1
The remaining von Neumann entropy of a black hole	Mode 2 takes the extreme value
The black hole first emits one of the entangled photons	The vacuum fluctuation causes the radiation particles in the entangled pair to cross the cutoff surface
All entangled photon pairs have been separated	The results of mode 1 and mode 2 are equal
The radiation portion begins to fully contain entangled photon pairs	How to take the extreme value of mode 2

**Figure A1:**
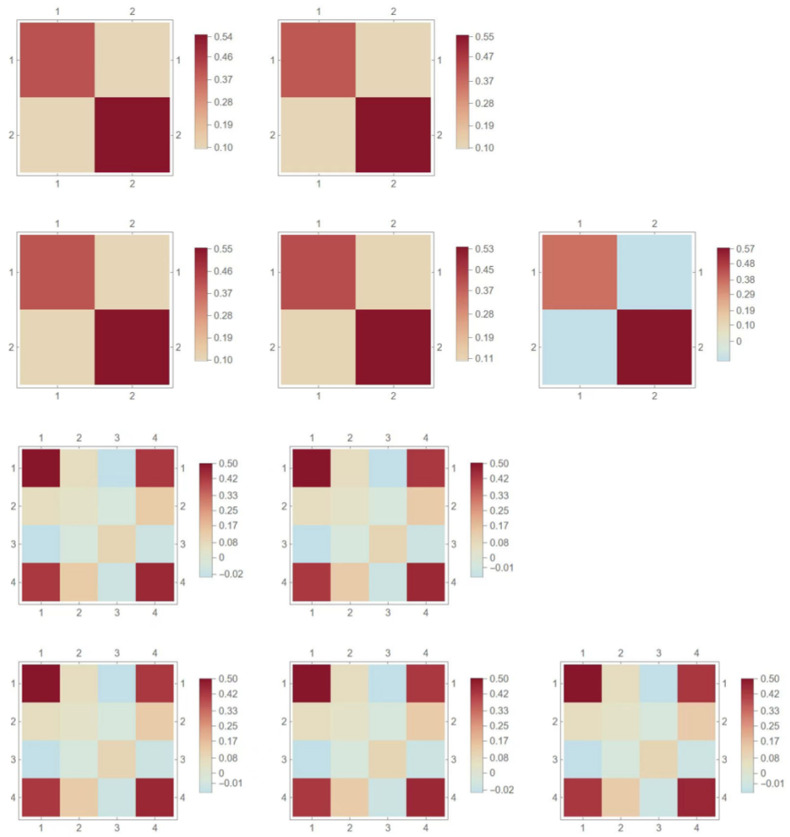
The density matrix obtained through quantum state tomography based on the first set of data. The first figure is obtained from the data of H-1 to L-1, the sixth figure is derived from the data of HH-6 to DV-6, and the other figures follow the same pattern.

**Figure A2:**
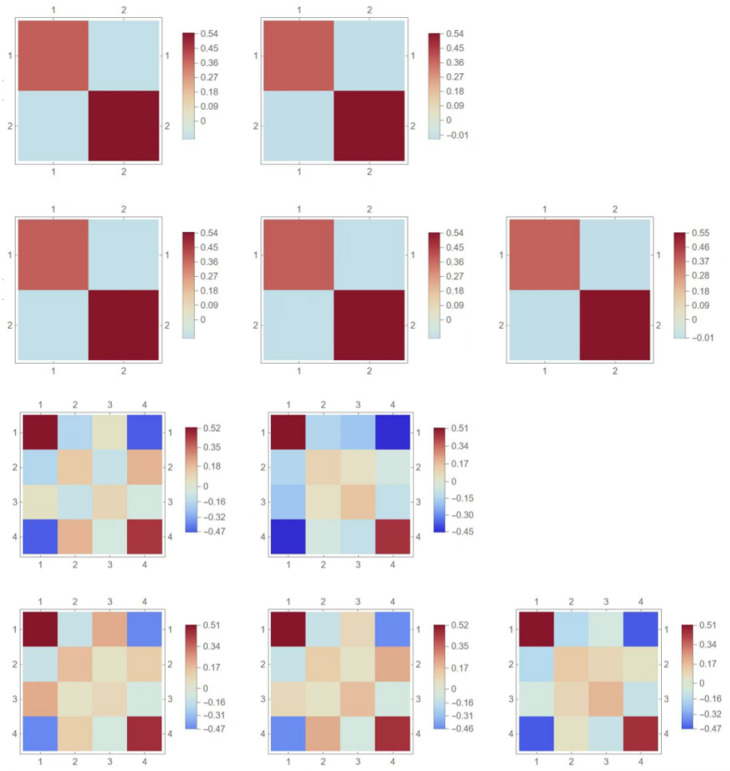
The density matrix obtained through quantum state tomography based on the second set of data.

**Figure A3:**
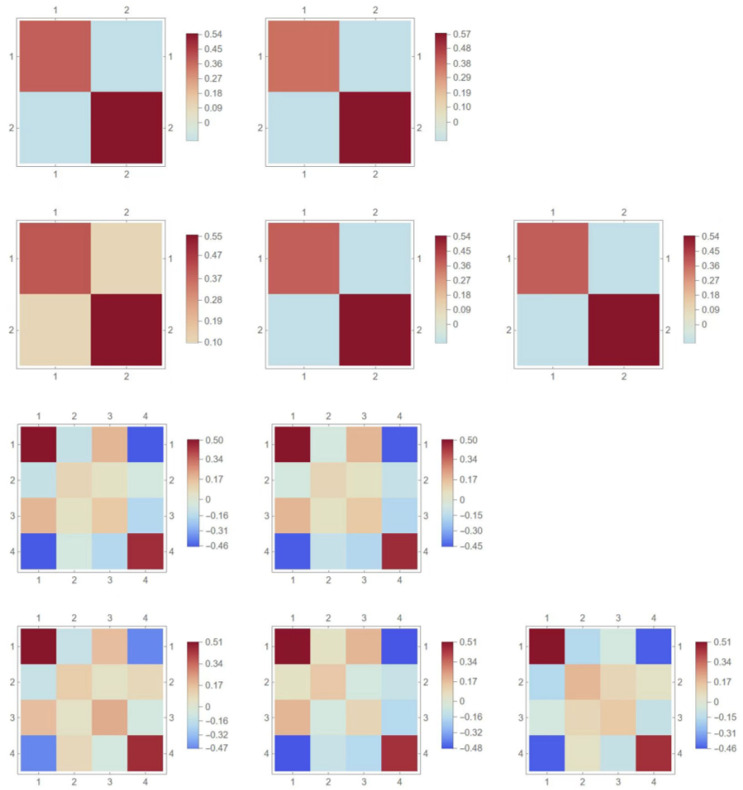
The density matrix obtained through quantum state tomography based on the third set of data.

## Data Availability

The data that support the findings of this study are available from the corresponding author upon request.

## Appendix A. Derivation Process of the Theoretical Model

The Unruh Effect means that an observer accelerating in a vacuum can feel an additional background temperature. In natural units, this temperature is related to acceleration by

(A1) $$T=\frac{a}{2\pi }.$$

To determine this temperature, it is necessary to calculate the corresponding acceleration. This involves selecting a stationary reference point located near the event horizon $\left(dx^{i}=0\right)$:

(A2) $$r=2M+\frac{\rho^{2}}{8M},$$

where ρ is a small amount, and we can obtain

(A3) $$\begin{array}{cc} 1-\frac{2M}{r} & =\frac{2m+\frac{\rho^{2}}{8M}-2M}{2M+\frac{\rho^{2}}{8M}}\\ \; & =\frac{\rho^{2}}{16M^{2}}\cdot \frac{1}{1+\frac{\rho^{2}}{16M^{2}}}\\ \; & =\frac{\rho^{2}}{16M^{2}}+o\left(\rho^{4}\right). \end{array}$$

Under the natural system of units, the metric of the Schwarzschild black hole is

(A4) $${\left(ds\right)}^{2}=-\left(1-\frac{2M}{r}\right){\left(dt\right)}^{2}+\frac{1}{1-\frac{2M}{r}}{\left(dr\right)}^{2}+r^{2}{\left(d\Omega \right)}^{2},$$

and we can obtain

(A5) $$\begin{array}{cc} {\left(ds\right)}^{2} & =-{\left(\frac{\rho }{4M}\right)}^{2}{\left(dt\right)}^{2}+{\left(\frac{\rho }{4M}\right)}^{2}{\left[d\left(2M+\frac{\rho^{2}}{8M}\right)\right]}^{2}+{\left(2M+\frac{\rho^{2}}{8M}\right)}^{2}{\left(d\Omega \right)}^{2}\\ \; & =-{\left(\frac{\rho }{4M}\right)}^{2}{\left(dt\right)}^{2}+{\left(d\rho \right)}^{2}+r^{2}{\left(d\Omega \right)}^{2}\\ \; & =-{\left(\frac{\rho }{4M}\right)}^{2}{\left(dt\right)}^{2}+{\left(d\rho \right)}^{2}+{\left(dX_{⊥}\right)}^{2}. \end{array}$$

The metric after the coordinate transformation is

(A6) $$g_{\mu \nu }=\left(\begin{array}{ccc} -{\left(\frac{\rho }{4M}\right)}^{2} & \; & \\ \; & 1 & \\ \; & \; & 1. \end{array}\right)$$

Since the selected point is a stationary reference point, the four speeds have only the time component, and the definition of the reuse metric can be obtained:

(A7) $$u^{\alpha }u_{\alpha }=g_{\mu \nu }\frac{dx^{\mu }}{d\tau }\frac{dx^{\nu }}{d\tau }=-{\left(\frac{\rho }{4M}\right)}^{2}u^{0}u^{0}=-c^{2}=-1.$$

Therefore, the fourth speed is

(A8) $$u^{\alpha }=\left(\frac{4M}{\rho },0,0,0\right).$$

According to the definition of the covariant acceleration,

(A9) $$a^{\mu }=\frac{\nabla u^{\mu }}{d\tau }=\frac{du^{\mu }}{d\tau }+\Gamma_{\nu \lambda }^{\mu }u^{\nu }u^{\lambda }=\Gamma_{00}^{\mu }u^{0}u^{0}.$$

$\Gamma_{00}^{\rho }=\frac{\rho }{{\left(4M\right)}^{2}}$ is not 0. The acceleration obtained after insertion is

(A10) $$a^{\rho }=\frac{\rho }{{\left(4M\right)}^{2}}\frac{4M}{\rho }\frac{4M}{\rho }=\frac{1}{\rho }.$$

So, the temperature near a point outside of the event horizon is

(A11) $$T=\frac{a}{2\pi }=\frac{1}{2\pi \rho }=\frac{1}{4\pi \sqrt{2Mr\left(1-\frac{2M}{r}\right)}}.$$

Considering the gravitational red-shift effect, the temperature at the r′ is

(A12) $$T\left(r\prime \right)=T\left(r\right)\sqrt{\frac{1-2M/r}{1-2m/r\prime }}=\frac{1}{4\pi \sqrt{2mr\left(1-\frac{2M}{r\prime }\right)}}.$$

If we take r′→∞, we obtain get the Hawking temperature of the black hole:

(A13) $$T\left(\infty \right)=\frac{1}{8\pi M}.$$

For a blackbody radiation model with known temperature, the energy radiated per unit time, per unit surface area, and per unit frequency interval is given by

(A14) $$\begin{array}{cc} I(\nu ,T)d\nu & =\frac{2h\nu^{3}}{c^{2}}\frac{1}{e^{h\nu /k_{B}T}-1}d\nu \\ \; & =4\pi \nu^{3}\frac{1}{e^{2\pi \nu /T}-1}d\nu . \end{array}$$

The corresponding particle number distribution is

(A15) $$\begin{array}{cc} N(\nu ,T) & =\frac{I(\nu ,T)}{h\nu }d\nu =\frac{2\nu^{2}}{c^{2}}\frac{1}{e^{h\nu /k_{B}T}-1}d\nu \\ \; & =2\nu^{2}\frac{1}{e^{2\pi \nu /T}-1}d\nu . \end{array}$$

By integrating the frequency, the energy and number of particles per unit time and unit surface area are

(A16) $$\begin{array}{cc} \; & \begin{array}{cc} p & =\int_{0}^{+\infty }I\left(\nu ,T\right)d\nu =\int_{0}^{+\infty }4\pi \nu^{3}\frac{1}{e^{2\pi \nu /T}-1}d\nu \\ \; & =4\pi {\left(\frac{T}{2\pi }\right)}^{4}\int_{0}^{\infty }x^{3}\frac{1}{e^{x}-1}dx\\ \; & =AT^{4} \end{array}\\ \; & \begin{array}{cc} n & =\int_{0}^{+\infty }N\left(\nu ,T\right)d\nu =\int_{0}^{+\infty }2\nu^{2}\frac{1}{e^{2\pi \nu /T}-1}d\nu \\ \; & =BT^{3}. \end{array} \end{array}$$

The coefficient is

(A17) $$\begin{array}{cc} A & =\frac{\pi }{60}\\ B & \approx 2.40411\times \frac{1}{4\pi^{3}}. \end{array}$$

The event horizon $\left(r_{0}=2M\right)$ was used as the radiation surface and brought in (A13). The total energy and total number of particles emitted per unit time are

(A18) $$\begin{array}{cc} \; & P=4\pi r_{0}^{2}\cdot p=C_{p}M^{-2}\\ \; & N=4\pi r_{0}^{2}\cdot n=C_{N}M^{-1}. \end{array}$$

The coefficient is

(A19) $$\begin{array}{cc} C_{p} & =\frac{A}{256\pi^{3}}=\frac{1}{15360\pi^{2}}\\ C_{N} & =\frac{B}{32\pi^{2}}\approx \frac{2.40411}{128\pi^{5}}. \end{array}$$

## Appendix B. Phase Modulation

In the experiment, a photon pair in a maximally entangled state was prepared using a two-photon entanglement source. To ensure that the target state was maximally entangled, phase modulation of the quantum state was applied (taking the $|\varphi \rangle =\frac{1}{\sqrt{2}}(|HH\rangle +\left|VV\rangle \right)$ state as an example). The letters H and V represent the horizontal and vertical directions, respectively. The quantum state of the photon in the H polarization direction is $|H\rangle =\left(\begin{array}{c} 1\\ 0 \end{array}\right)$.

The photon quantum state in the polarization direction of V is written as $|V\rangle =\left(\begin{array}{c} 0\\ 1 \end{array}\right)$. In addition to the horizontal (H) and vertical (V) measurement bases used in the modulation and measurement process, there are also oblique 45 degree polarization measurement ground states A($|A\rangle =\frac{1}{\sqrt{2}}(|H\rangle +\left|V\rangle \right)$) and D($|D\rangle =\frac{1}{\sqrt{2}}(|H\rangle -\left|V\rangle \right)$) and left and right rotation polarization measurement bases L($|L\rangle =\frac{1}{\sqrt{2}}(|H\rangle +i|V\rangle )$) and R($|R\rangle =\frac{1}{\sqrt{2}}(|H\rangle -i|V\rangle )$).

Since the BBO crystal will add a phase when producing entangled photon pairs [27,28], the resulting quantum state is $|\psi \rangle =|HH\rangle +e^{i\beta }|VV\rangle$. To eliminate this relative phase, two half-wave plates and two quarter-wave plates were added in front of the BBO crystal. When the angle between the optical axis and the horizontal polarization direction (*H*) of the half-wave plate and the quarter-wave plate was θ, their evolution operator was

(A20) $$U_{HWP}\left(\theta \right)=\left(\begin{array}{cc} \cos2\theta & \sin2\theta \\ \sin2\theta & -\cos2\theta \end{array}\right)$$

(A21) $$U_{QWP}\left(\theta \right)=\left(\begin{array}{cc} \cos^{2}\theta +i\sin^{2}\theta & (1-i)\cos\theta \sin\theta \\ (1-i)\cos\theta \sin\theta & \;i\cos^{2}\theta +\sin^{2}\theta \end{array}\right).$$

Let the initial state of the photon be *H*, and before passing through the BBO crystal, it first passes through a half-wave plate with its optical axis is oriented at π/4. Then, a quarter-wave plate with with its optical axis is also oriented at π/4, Next, the photon passes through an unknown direction of α and finally through another half-wave plate with its optical axis oriented at π/8. Then, the quantum state of the photon becomes

(A22) $$\begin{array}{cc} |\psi_{1}\rangle & =U_{QWP}\left(\pi /4\right)U_{HWP}\left(\alpha \right)U_{QWP}\left(\pi /4\right)U_{HWP}\left(\pi /8\right)|H\rangle \\ \; & =\left(\begin{array}{c} \frac{i}{\sqrt{2}}e^{-2i\alpha }\\ -\frac{i}{\sqrt{2}}e^{2i\alpha } \end{array}\right)=\left(\begin{array}{c} \frac{1}{\sqrt{2}}\\ -\frac{1}{\sqrt{2}}e^{4i\alpha } \end{array}\right)e^{-2i\alpha }. \end{array}$$

At this point, after passing through the BBO crystal, the quantum state acquires an additional phase β. Discarding the global phase factor $e^{-2i\alpha }$, the quantum state becomes

(A23) $$|\psi_{2}\rangle =\left(\begin{array}{c} \frac{1}{\sqrt{2}}\\ 0\\ 0\\ \frac{1}{\sqrt{2}}e^{i(4\alpha +\beta )} \end{array}\right).$$

In order to make the relative phase 0, we need to adjust the angle of the half plate α to offset the extra phase brought by the BBO crystal. Coincidence counting is measured under the DD base vector. Theoretically, coincidence counting satisfies the following relationship:

(A24) $$|\langle DD|\psi_{2}\rangle {|}^{2}=\frac{1}{4}\left(1+\cos(4\alpha +\beta )\right).$$

It can be seen that when the relative phase value is 0, the coincidence count value under the DD measurement base is the largest. Therefore, we adjusted the half wave plate α, observed the coincidence count under the DD measurement base, and adjusted it to the maximum; then, we confirmed that the relative phase was 0. Similarly, to achieve the target state $|\varphi \rangle =\frac{1}{\sqrt{2}}(|HH\rangle -\left|VV\rangle \right)$, one can simply adjust until the coincidence count in the DD basis reaches its minimum.

## Appendix C. Raw Experimental Data

The experimental raw data are presented below, with the values in each table representing averages. For example, the data for single channel 1-*H* in the table are derived by averaging 10 consecutive real-time measurements from the *H* measurement basis. In this study, three datasets were collected, corresponding to the first, second, and third tables, respectively.

The data were analyzed using quantum state tomography as described in Section 4. In calculating $P_{|H\rangle }$, the total photon number is taken as $n_{|H\rangle }$+$n_{|V\rangle }$; so, $P_{|H\rangle }=\frac{n_{|H\rangle }}{n_{|H\rangle }+n_{|V\rangle }}$. When calculating the probability of coincidence counting, we used $n_{|HH\rangle }$+$n_{|HV\rangle }$+$n_{|VH\rangle }$+$n_{|VV\rangle }$ as the total count. For example, $P_{|HH\rangle }$=$\frac{n_{|HH\rangle }}{n_{|HH\rangle }+n_{|HV\rangle }+n_{|VH\rangle }+n_{|VV\rangle }}$. The single-photon density matrix was reconstructed through photon counting, while the entangled state density matrix was reconstructed via coincidence counting, with the results illustrated in Figure 3a,b.

The von Neumann entropy was computed from the density matrix, and the time evolution of two-photon entanglement in the Hawking radiation model was determined using Equation (10) from the paper, as shown in Figure 3c.

**Table A1:** The first set of raw experimental data.

Measurement Bases	Photon Counts				
	**1**	**2**	**3**	**4**	**5**
H	23,268	23,350	23,008	23,105	22,448
V	27,862	27,710	27,534	27,430	27,560
D	25,288	24,669	25,032	25,243	25,150
A	25,536	25,929	25,880	25,969	26,192
R	25,379	25,316	25,861	25,008	25,727
L	24,777	24,882	24,875	24,822	24,363
**Coincidence Counts**					
	**6**	**7**	**8**	**9**	**10**
HH	3727	3715	3710	3735	3709
HV	31	21	24	21	25
HR	2019	2024	2026	2028	2006
HD	2183	2180	2183	2217	2179
VD	1443	1427	1429	1448	1460
VR	1764	1777	1779	1792	1786
VH	80	79	84	80	82
VV	3622	3614	3643	3608	3627
RV	2069	2062	2062	2059	2026
RH	1854	1888	1889	1870	1890
RR	3885	3901	3900	3876	3869
RD	2021	2052	2057	2037	2047
DD	62	66	70	64	63
DR	2288	2307	2298	2314	2283
DH	2351	2331	2336	2332	2328
DV	1872	1866	1881	1897	1866

**Table A2:** The second set of raw experimental data.

Measurement Bases	1	2	3	4	5
**Photon Counts**					
H	26,099	25,460	25,582	25,736	25,079
V	31,637	31,998	31,769	29,986	34,153
D	29,048	29,248	29,367	28,180	31,228
A	28,212	28,428	28,541	25,818	32,212
L	29,086	29,428	29,542	28,244	32,409
R	29,458	29,603	29,209	28,081	32,263
**Coincidence Counts**					
	6	7	8	9	10
HH	3464	3376	3366	3540	3640
HV	28	32	13	22	11
HR	1672	1838	1782	1746	1716
HD	1995	2068	2059	1908	2107
VD	1602	1640	1676	1615	1656
VR	1660	1702	1752	1659	1634
VH	15	34	15	14	13
VV	3041	3216	3315	3496	3489
RV	1820	1738	1716	1725	1732
RH	1918	1775	1828	1888	1914
RR	47	54	51	38	51
RD	1814	1846	1832	1976	1838
DD	3636	3638	3647	3440	3575
DR	1890	1900	1964	1939	2004
DH	1740	1838	1744	1732	1698
DV	1864	2000	1925	1860	1869

**Table A3:** The third set of raw experimental data.

Measurement Bases	Photon Counts				
	**1**	**2**	**3**	**4**	**5**
H	22,448	23,048	22,934	23,280	22,922
V	27,662	27,510	27,435	27,408	27,455
D	24,788	24,910	24,897	25,612	25,088
A	25,909	25,204	26,146	26,016	25,695
R	25,020	25,624	25,500	25,250	25,430
L	24,640	24,731	24,722	25,076	24,960
**Coincidence Counts**					
	**6**	**7**	**8**	**9**	**10**
HH	3482	3595	3562	3434	3592
HV	18	19	18	20	18
HR	1734	1834	1735	1850	1765
HD	2062	1941	1986	1957	1922
VD	1583	1758	1613	1562	1687
VR	1684	1606	1670	1684	1665
VH	20	24	20	14	18
VV	3616	3584	3498	3465	3474
RV	1653	1632	1757	1644	1778
RH	1943	1874	1905	1856	1817
RR	44	49	26	33	50
RD	1844	1798	1862	1864	1814
DD	3616	3344	3518	3680	3710
DR	1890	1992	1879	1990	1983
DH	1706	1627	1854	1741	1794
DV	1914	1963	1888	1949	1852

## Appendix D. Maximum Likelihood Estimation Method

For measurement data comprising a set of 16 coincidence counts, it is assumed that the experimental errors associated with these coincidence measurements follow a normal distribution. If the quantum state is assumed to be ρ, the likelihood function is the probability of measuring the actual result

(A25) $$\begin{array}{cc} L & =P\left(n_{1},n_{2},\ldots ,n_{16}\right)=\frac{1}{N_{norm}}\prod_{s=1}^{16}\exp\left[-\frac{{\left(n_{s}-{\bar{n}}_{s}\right)}^{2}}{2{\bar{n}}_{s}}\right] \end{array},$$

where $\frac{1}{N_{norm}}$ is the normalization constant, $n_{s}$ represents the 16 coincidence counts measured under 16 measurement bases, and their expected value is ${\bar{n}}_{s}$.

To ensure the positivity of the density matrix, the state can be represented in the following form:

(A26) $$\hat{\rho }\left(t\right)={\hat{T}}^{†}\left(t\right)\hat{T}\left(t\right)/\mathrm{Tr}\left\{{\hat{T}}^{†}\left(t\right)\hat{T}\left(t\right)\right\}.$$

$\hat{T}\left(t\right)$ is the triangular matrix

(A27) $$\hat{T}\left(t\right)=\left(\begin{array}{cccc} t_{1} & 0 & 0 & 0\\ t_{5}+it_{6} & t_{2} & 0 & 0\\ t_{11}+it_{12} & t_{7}+it_{8} & t_{3} & 0\\ t_{15}+it_{16} & t_{13}+it_{14} & t_{9}+it_{10} & t_{4} \end{array}\right)$$

To simplify the calculation of the optimum value of the likelihood function, we take the negative logarithm of the likelihood function and substitute the relationship between the coincidence notation and the quantum state ${\bar{n}}_{s}=N\langle \psi_{s}\left|\hat{\rho }\left(t\right)\right|\psi_{s}\rangle$ (N here is taken as the total sum of the coincidence counts in the |HH〉, |HV〉, |VH〉, and |VV〉 bases) into the likelihood function to derive a new likelihood function, thereby transforming the problem into finding the minimum value of the new likelihood function

(A28) $$\begin{array}{c} L\left(t\right)=\sum_{s=1}^{16}\frac{{\left[N\langle \psi_{s}\left|\hat{\rho }\left(t\right)\right|\psi_{s}\rangle -n_{s}\right]}^{2}}{2N\langle \psi_{s}\left|\hat{\rho }\left(t\right)\right|\psi_{s}\rangle }. \end{array}$$

A set of initial values is derived from the non-physical density matrix obtained from Formula (13) [22], and then the minimum of the likelihood function is found through numerical calculations. Finally, the optimal T-matrix is determined, and the final density matrix is obtained using Formula (A26). References
